# Transcriptome and Metabolome Analysis of Low-Pressure Regulation in *Saussurea involucrata* Leaves

**DOI:** 10.3390/genes16030328

**Published:** 2025-03-11

**Authors:** Xinyu Quan, Fenggui Fan, Hanbo Cao, Na Tang, Changgen Xu, Changhe Wang

**Affiliations:** 1 Co-Construction Collaborative Innovation Center for Chinese Medicine, Resources Industrialization by Shaanxi & Education Ministry, Shaanxi University of Chinese Medicine, Xianyang 712083, China; xy072898@163.com; 2 Shaanxi Institute for Food and Drug Control, Xi’an 710038, China; hannecao@163.com (H.C.); tnlb2000@126.com (N.T.); changgenxu@126.com (C.X.); 3 Shaanxi Medical Devices Quality Testing Institute, Xixian New Area, Xianyang 712046, China

**Keywords:** *S. involucrata*, transcriptomics, hypobaric hypoxia, metabolomics, flavonoid regulation, oxidative stress

## Abstract

*Saussurea involucrata*, an endangered medicinal plant, thrives in high mountain regions at altitudes ranging from 3500 to 5000 m. Being a plant that grows at high altitudes means it possesses unique physiological mechanisms and stress-responsive genes that regulate and adapt to the high-altitude environment. While many cold-resistant genes have been cloned and their mechanisms studied, the genes and molecular mechanisms involved in adaptation to hypobaric hypoxia remain largely unexplored. This study conducted transcriptomic and metabolomic analyses on the leaves of *S. involucrata* under normal atmosphere (101 kPa) and low pressure (60 kPa). A total of 2383 differentially expressed genes (DEGs) and 336 differentially accumulated metabolites (DAMs) were identified utilizing RNA-seq and UPLS-MS techniques. The results indicated that *S. involucrata* exhibits responses to hypobaric hypoxia environments by engaging in DNA repair, membrane transport, hypoxic response, reproductive processes, and various metabolic activities associated with nutrient uptake and the effective utilization of chemical components. It is worth noting that under low-pressure treatment, flavonoids are predominantly negatively regulated, whereas terpenoids are primarily positively regulated. These findings identify key genes and metabolites in *S. involucrata* that respond to hypobaric hypoxia treatment, providing a theoretical basis for the development of its medicinal value and for low-altitude cultivation.

## 1. Introduction

A hypobaric hypoxia environment is one of the major environmental stressors that plants encounter for growth and development at high altitudes. This condition can affect plant physiological and biochemical processes by altering the rates of gas diffusion and solubility [1]. Under optimal temperature, humidity, and light conditions, low atmospheric pressure treatment of various plants significantly reduces growth and proliferation. However, it simultaneously enhances the accumulation of bioactive components, as well as respiration and photosynthesis, thereby protecting the plants from reactive oxygen species (ROS) damage. These observations suggest that plants possess genetic mechanisms to adapt to low atmospheric pressure [2,3]. The growth and developmental status of plants varies across different hypobaric hypoxia environments, influencing both accumulated biomass and gene expression. Evaluation of the model plant *A. thaliana* has shown that adaptation to hypoxia involves a significantly higher activation of metabolic pathways in branches exposed to either hypoxia or anoxia compared to those activated solely for hypoxia adaptation [3]. Plants can modify their development in response to hypoxic stress to enhance oxygen transport. Acclimation to hypoxic environments involves the formation of gas-filled aerated tissues and the initiation of adventitious roots, processes that are regulated through the ethylene pathway [4]. The DNA Microarray Report identifies genes, such as heat shock proteins and ethylene-responsive binding proteins, that respond to hypoxia mechanisms in A. thaliana [5,6,7]. Research has shown that plants growing in high-altitude environments tend to remodel and reconfigure their transcriptomes to adapt to climate variability [8]. Alpine species enhance their low-oxygen adaptability by recruiting more oxidoreductase-related genes [9], activating enzymes and non-enzyme antioxidants, including superoxide dismutase (SOD), catalase (CAT), ascorbate peroxidase (APX), and glutathione reductase (GR) [10,11,12]. In addition, based on metabolomic analysis, it was found that *Saussurea* (*Asteraceae*) adapts to high-altitude environments by up-regulating phenylpropanoid biosynthesis and phenylalanine metabolism in its leaves [9]. However, it remains unclear whether the activation of these metabolic pathways is directly regulated by the low-pressure environment. Further elucidation of the molecular mechanisms is necessary through low-pressure control experiments in conjunction with multi-omics analyses.

*S. involucrata* is a plant that thrives in high-altitude regions, possessing unique physiological mechanisms to regulate and adapt to hypobaric hypoxia environments. This species belongs to the *Asteraceae family* and the genus *Saussurea* [13]. It is a perennial dicotyledonous alpine herb recognized for its significant medicinal value. Modern pharmacological research indicates that *S. involucrata* can effectively alleviate fatigue [14], provide antioxidant effects [14], treat cancer [13], reduce inflammation [15], protect cardiac health [16], and address brain injuries [17]. *S. involucrata* contains flavonoids, phenolic compounds, polysaccharides, and terpenoids [18], and its health benefits are directly linked to its phytochemical composition [19,20]. However, due to illegal harvesting and climate change, the resources of *S. involucrata* have sharply declined without receiving appropriate and effective protection [21]. Research has demonstrated that the overexpression of *S. involucrata* fruit sugar 1,6-bisphosphate aldolase (*SiFBA5*) enhances the cold tolerance and photosynthetic efficiency of transgenic tomatoes [22]. The *S. involucrata* dehydrin gene (*SiDHN*) improves the stress responses of transgenic tomato plants by maintaining cell membrane integrity, reducing chlorophyll photo-oxidation and the accumulation of reactive oxygen species, enhancing antioxidant enzyme activity and photochemical electron transfer efficiency [23]. The overexpression of *S. involucrata* metal proteinase (*SikCuZnSOD3*) increases the abiotic stress resistance of cotton by boosting antioxidant enzyme activity, maintaining reactive oxygen species homeostasis, and minimizing cell membrane damage [24]. Moreover, *S. involucrata* exhibits resistance to drought, low atmospheric pressure, and strong UV radiation, making it a valuable resource for studying plant adaptation to various abiotic stressors.

Transcriptomics is a crucial method for studying gene expression, enabling quantitative analysis of gene expression levels, identification of differentially expressed genes, and elucidation of gene regulatory networks. In contrast, metabolomics focuses on examining the types and quantities of metabolites in organisms under specific conditions, utilizing techniques such as UPLC-MS and GC-MS to identify, quantify, and analyze changes in metabolic pathways. The combined application of transcriptomics and metabolomics facilitates the construction of gene–metabolite networks, discovery of key regulatory nodes, and unveiling of the comprehensive regulatory chain linking genes to metabolites, thereby providing a more thorough understanding of the molecular response mechanisms of organisms under specific conditions. Recently, transcriptome technology has been extensively utilized to investigate the mechanisms of hypobaric hypoxia in various species, including *Arabidopsis thaliana*, maize, tomato, and gray poplar [3,25,26,27]. These studies lay the groundwork for a more profound understanding of the adaptation mechanisms of plants to hypoxic environments. Previously, the selection of adversity resistance genes in *S. involucrata* was primarily focused on its resistance to cold and low temperatures. However, the molecular mechanisms that allow *S. involucrata* to adapt to hypobaric hypoxia environments remain unclear. Therefore, understanding the adaptation mechanisms of *S. involucrata* under varying atmospheric pressure treatments and exploring genes associated with low-pressure regulation are of significant importance for conservation and domestication. In this study, we subjected the leaves of *S. involucrata* to normobaric normoxic (NN: 101 kPa) and hypobaric hypoxia (HH: 60 kPa) treatments, followed by a comprehensive analysis of the transcripts and metabolites. The key DEGs involved in antioxidant defense response, hypoxia adaptation, stress response, and metabolic changes under low pressure were identified. KEGG enrichment analysis indicated that these DEGs and DAMs were significantly enriched in flavonoid synthesis and linolenic acid metabolic pathways. These results elucidate the key pathways regulated by low pressure in *S. involucrata*, providing a foundation for low-altitude cultivation and resource conservation of this species.

## 2. Results

### 2.1. Phenotypic Changes in S. involucrata Leaves Under Different Air-Pressure Treatments

**Figure 1:**
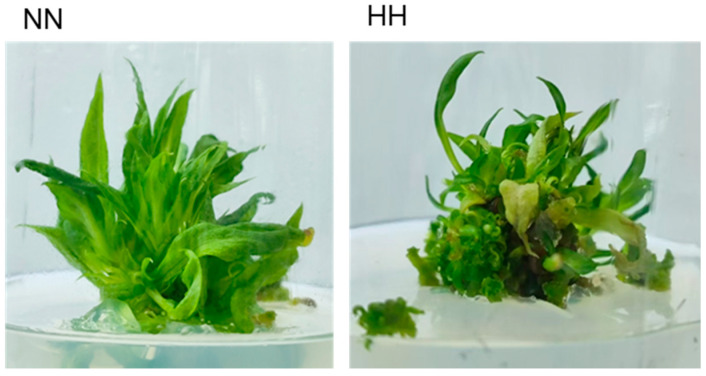
Phenotypic observations of *S. involucrata* in the NN group and HH group after three weeks of treatment: **left**: normobaric normoxic, **right**: hypobaric hypoxia.

The tissue culture seedlings of *S. involucrata* were subjected to treatments at atmospheric pressures of 60 kPa and 101 kPa for a duration of three weeks. Observations indicate that the seedlings exhibited superior growth under normal-pressure conditions. In contrast, the leaves exposed to low pressure appeared dry and yellow compared to those treated under normal pressure. Additionally, there was a higher occurrence of callus formation, and the differentiation rate of buds was inferior to that of the tissue culture seedlings treated at 101 kPa (Figure 1).

### 2.2. Transcriptome Analysis of the S. involucrata

#### 2.2.1. Transcriptome Data-Quality Assessment of the *S. involucrata* and Identification of DEGs

**Figure 2:**
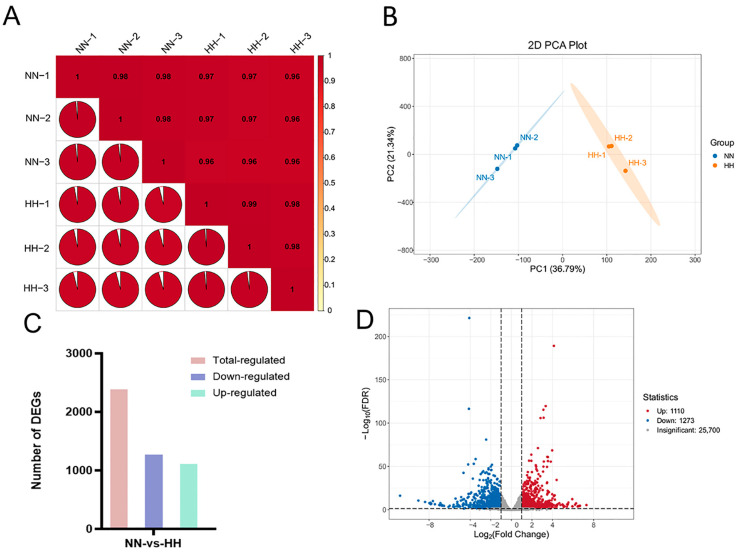
Transcriptome data-quality assessment and analysis of DEGs. (**A**) Pearson correlation test of 6 samples; (**B**) PCA of 6 samples, with different colors in the figure denoting distinct samples (NN: normobaric normoxic, HH: hypobaric hypoxia); (**C**) histogram of *S. involucrata* DEGs; (**D**) volcano map of DEGs in *S. involucrata*.

Total RNA was extracted from the leaves of *S. involucrata* subjected to different pressure treatments (101 kPa and 60 kPa). Quality assessment of the RNA revealed that the 5S, 18S, and 28S rRNA bands were intact, with no apparent degradation, indicating that the extracted RNA was of high quality (Supplementary Figure S1). A total of 43.77 Gb clean reads were obtained from transcriptome sequencing analysis of six samples, with each sample achieving a clean read count of 6.0 Gb. Q20 exceeded 98%, while the Q30 value surpassed 94% (Supplementary Table S1). Additionally, the proportion of GC content in the transcript of each sample reached 44%. The transcriptome data from six cDNA libraries were aligned with the genome of *S. involucrata*, resulting in mapped reads exceeding 83% for each sample (Supplementary Table S2). These results confirm the high quality of the RNA-Seq dataset, ensuring the reliability of subsequent analyses and meeting the criteria necessary for further bioinformatics investigation. Pearson correlation coefficients for all gene expression values were calculated both between samples and within samples, and the Pearson correlation among all processing replicates ranged from 0.96 to 0.99 (Figure 2A). This figure illustrates the correlation of gene expression in *S. involucrata* under NN and HH conditions. A higher correlation coefficient indicates greater similarity in gene expression. In the principal component analysis (PCA) plot, groups subjected to different baric treatments exhibited a tendency to cluster together. PC1 accounted for 36.79% of the variance, while PC2 accounted for 21.34% of the variance (Figure 2B).

The transcriptome of *S. involucrata*, obtained through various pressure treatments, revealed 2383 DEGs when comparing the two treatment groups, NN vs. HH. Among these, 1110 genes were identified as up-regulated, while 1273 genes were down-regulated (Figure 2C,D). We sorted the genes according to the value of log2FC and identified the first five up-regulated DEGs as novel.2199, SnowLotus14972, novel.2797, novel.432, and novel.1138. The first five down-regulated DEGs were novel.5883, novel.6353, novel.2793, novel.6352, and novel.2971 (Supplementary Table S3). Notably, with the exception of SnowLotus14972, the remaining nine genes were newly identified from the transcriptome, with the prediction based on the revealed structure identifying three genes encoding retrovirus-related Pol polyprotein derived from the transposon RE. Their expression was found to be down-regulated, potentially due to the increased *S. involucrata* resistance to stress resulting from the transactivation of the LTR transposon [28].

#### 2.2.2. Enrichment Analysis of DEGs

**Figure 3:**
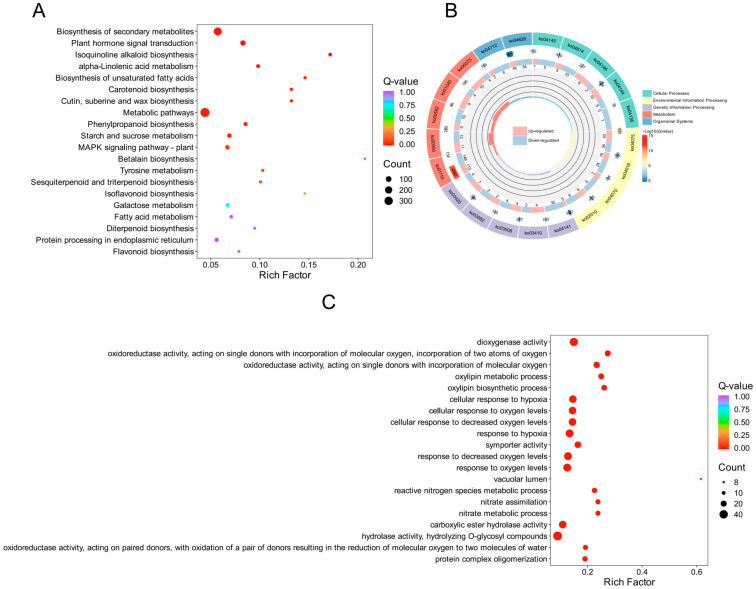
DEG enrichment analysis scatter plot and circle plot. (**A**) Scatter plot of KEGG enrichment analysis of DEGs; (**B**) circle plot of KEGG enrichment analysis of DEGs; (**C**) scatter plot of GO enrichment analysis of DEGs. The color of the dots indicates the enrichment significance, while the size of the dots reflects the number of DEGs that are enriched.

To elucidate the internal regulation of genes in *S. involucrata* under varying barometric pressure treatments, we generated two transcriptomes (NN and HH). KEGG enrichment analyses indicated that a total of 726 DEGs were significantly enriched in the KEGG pathways when comparing NN to HH. Among these, 365 DEGs were notably enriched in metabolic pathways, constituting the largest proportion at 50.28%. This enrichment may be attributed to the fact that *S. involucrata*, recognized as a traditional Chinese medicine, contains a diverse array of secondary metabolites. The DEGs related to starch and sucrose, phenylpropanoid, linolenic acid, galactose, isoquinoline alkaloids, fatty acids, sesquiterpenes, and triterpenes were significantly enriched across metabolic pathways. In the environmental information processing category, DEGs were primarily enriched in the plant hormone signal transduction and MAPK signaling pathways, with 77 and 40 DEGs identified in each category, respectively. These pathways are crucial for plant growth and development, indicating that a significant proportion of these genes may mitigate the detrimental effects of low-pressure hypoxia on the growth and development of *S. involucrata* by regulating key physiological processes, such as molecular synthesis, transport mechanisms, and cell wall synthesis and modification. Additionally, within the category of organismal systems, DEGs were significantly enriched in the plant–pathogen interactions, comprising a total of 73 genes. In the category of genetic information processing, protein processing in the endoplasmic reticulum was significantly enriched, with a total of 37 DEGs (Figure 3A and Figure S2). Approximately 1/3 of secreted and membrane proteins require proper folding and maturation within the ER. We hypothesize that a low-pressure hypoxic environment may impact the correct folding of these proteins. To further elucidate the regulation and function of DEGs within the KEGG pathway, we selected the KEGG-enriched pathway with the smallest q-value among the five categories. Our analysis revealed that the number of down-regulated DEGs exceeded that of up-regulated DEGs across all categories (Figure 3B).

After revealing the biological pathways and metabolic networks in which the genes were involved through KEGG enrichment analysis, we conducted GO functional annotation analysis to further elucidate the biological roles of these genes at the functional level. This analysis aimed to clarify the specific roles of the genes in molecular functions, biological processes, and cellular components. The results indicated that a total of 1246 DEGs were identified through GO enrichment analysis, and the 50 GO terms with the lowest *p*-values from the enrichment analysis were selected for visual analysis. In the biological process (BP) category, the primary entries included lipid catabolic process (GO: 0016042), terpenoid metabolic process (GO: 0006721), and fatty acid biosynthetic process (GO: 0006633), with the corresponding numbers of DEGs being 40, 39, and 37. Within the cellular component (CC) category, the number of DEGs identified in the vacuolar lumen (GO: 0005775) was eight. In the molecular function (MF) category, DEGs associated with hydrolase activity, specifically hydrolyzing O-glycosyl compounds (GO: 0004553), secondary active transmembrane transporter activity (GO: 0015291), and dioxygenase activity (GO: 0051213), were significantly enriched, with counts of 45, 39, and 38 DEGs, respectively (Figure 3C and Figure S3). It is worth noting that we revealed many pathways related to oxygen levels in GO enrichment analysis, including responses to hypoxia (GO: 000166), variations in oxygen levels (GO: 0070482), and decreased oxygen levels (GO: 0036293). The DEGs identified within these pathways predominantly pertain to metabolism and signal transduction, as indicated.

### 2.3. Metabolome Analysis of the S. involucrata

#### 2.3.1. Detection and Screening of DEGs

**Figure 4:**
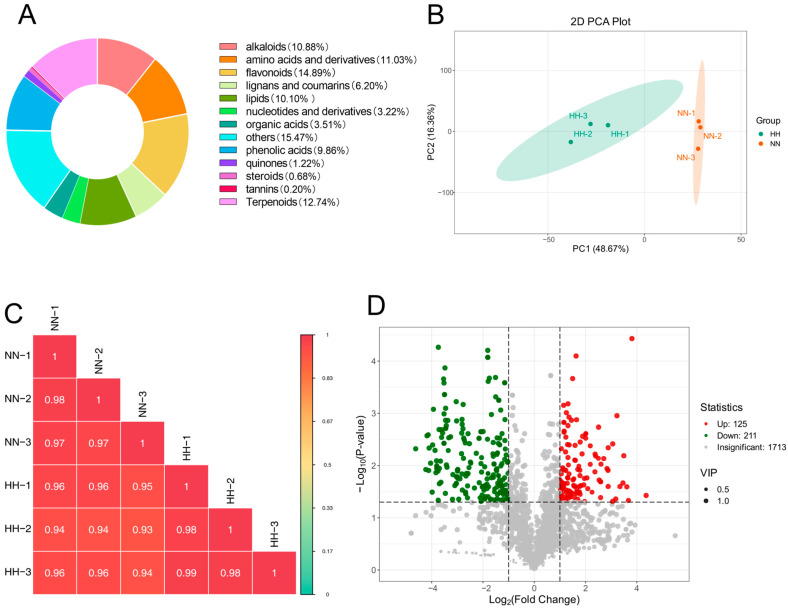
Assessment of metabolome data quality and analysis of DAMs. (**A**) The metabolite categories of *S. involucrata* are represented in a ring diagram. Each color corresponds to a specific metabolite category, with the area of each color block indicating the proportion of that category; (**B**) PCA of metabolites across 6 samples; (**C**) Pearson correlation test of *S. involucrata* treated with NN vs. HH for each metabolite expression level between the groups; (**D**) volcano plot of DAMs in *S. involucrata*.

To assess the effects of NN and HH treatments on the secondary metabolites of *S. involucrata*, the leaves of *S. involucrata* were detected using widely targeted metabolomics. A total of 2049 metabolites were identified across 6 samples, which were categorized into 13 distinct groups. The composition of these metabolites included flavonoids (14.89%), terpenoids (12.74%), amino acids and derivatives (11.03%), alkaloids (10.88%), lipids (10.1%), phenolic acids (9.86%), lignans and coumarins (6.2%), organic acids (3.51%), nucleotides and derivatives (3.22%), quinones (1.22%), steroids (0.68%), and tannins (0.2%) (Figure 4A).

To ensure the reliability of the analysis process, we assessed the biological repeatability among the samples in the observation group by analyzing the correlation. The Pearson correlation coefficient was calculated for both the samples and their respective metabolites. The results indicated that the repeatability within the group ranged from 0.97 to 0.99, while the repeatability between the groups ranged from 0.93 to 0.96, demonstrating good repeatability (Figure 4C). The results of the PCA indicated that the samples exhibited excellent repeatability across the groups. PC1 accounted for 48.67%, while PC2 accounted for 16.36% (Figure 4B). A total of 2049 metabolites were identified following LC-MS analysis of *S. involucrata* treated under NN and HH conditions. DAMs were defined using the criteria of |log2FC| > 1 or *p* < 0.05, along with a variable importance projection (VIP) > 1. This analysis yielded 336 differential metabolites, comprising 125 up-regulated DAMs and 211 down-regulated DAMs (Figure 4D). The number of down-regulated DAMs exceeded that of up-regulated DAMs, which aligns with the transcriptome results. This observation may be attributed to the oxidative stress induced by low-pressure treatment, potentially inhibiting the expression of certain genes and metabolites.

#### 2.3.2. KEGG Enrichment Analysis of DAMs

**Figure 5:**
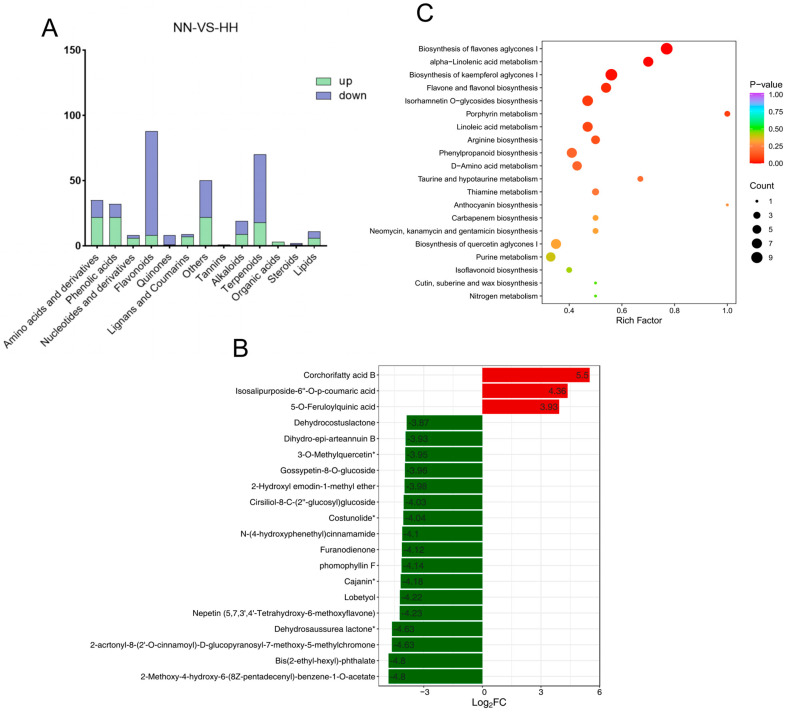
Scatterplot of DAM screening and KEGG enrichment analysis. (**A**) The types of DAMs in *S. involucrata* under NN vs. HH treatment. Blue indicates down-regulated DAMs, while green denotes up-regulated DAMs. (**B**) The top 20 DAMs from the *S. involucrata* leaves. The horizontal axis represents the log2FC of the differential metabolites, and the vertical axis displays the differential metabolites. (**C**) KEGG pathway enrichment of *S. involucrata,* Where the symbol superscripted after the compound means that the substance has isomers.

Compared to NN conditions, the levels of flavonoids, quinones, terpenoids, and steroids in the leaves of *S. involucrata* subjected to HH decreased. In contrast, the concentrations of amino acids and derivatives, phenolic acids, nucleotides and their derivatives, lignans, coumarins, and organic acids increased (Figure 5A). Therefore, it can be inferred that the metabolite profiles of *S. involucrata* vary under different air-pressure treatments, which may influence its therapeutic effects and medicinal efficacy. In the NN vs. HH comparison, the first 20 metabolites with the largest absolute values of Log2FC were identified, revealing that only three compounds exhibited increased content. This indicates that relative to NN conditions, the reduction in compound content following HH treatment was more pronounced than the increases observed. The up-regulated compounds included corchorifatty acid B, isosalipurposide-6”-O-p-coumaric acid, and 5-O-feruloylquinic acid, while the down-regulated compounds comprised 2-methoxy-4-hydroxy-6-(8Z-pentadecenyl)-benzene-1-O-acetate, Bis(2-ethyl-hexyl)-phthalate, 2-acrtonyl-8-(2’-O-cinnamoyl)-D-glucopyranosyl-7-methoxy-5-methylchromone, and dehydrosaussurea lactone (Figure 5B). Dehydrosaussurea lactone, which is specific to *Saussurea,* was also found to be significantly down-regulated. Notably, the three up-regulated compounds were phenolic acids, while the 17 down-regulated compounds were predominantly terpenes and flavonoids, thereby corroborating previous findings.

To further elucidate the role and primary signaling pathways of DAMs in response to HH conditions, KEGG and MetMap enrichment analyses were conducted on the identified DAMs. A total of 85 pathways were implicated in the treatment of HH, with the differentially expressed metabolites primarily associated with metabolism, environmental information processing, and genetic information processing. Among them, the metabolic pathway contained the highest number of compounds. The top 20 pathways exhibiting the smallest *p* values were selected for display (Figure 5C). The metabolic pathways highlighted include the biosynthesis of flavone aglycones I (MetMap110), alpha-linolenic acid metabolism (Ko00592), the biosynthesis of kaempferol aglycones I (MetMap113), flavone and flavonol biosynthesis (Ko00944), porphyrin metabolism (Ko00860), linoleic acid metabolism (Ko00591), arginine biosynthesis (Ko00220), and phenylalanine metabolism (Ko00360). Overall, following various pressure treatments, the metabolic pathways associated with the enrichment of low-pressure and low-oxygen conditions were shown to involve a maximum of 53 DAMs.

### 2.4. Combined Analysis of the DEGs and DAMs

#### 2.4.1. DEG–DAM Correlation Analysis

**Figure 6:**
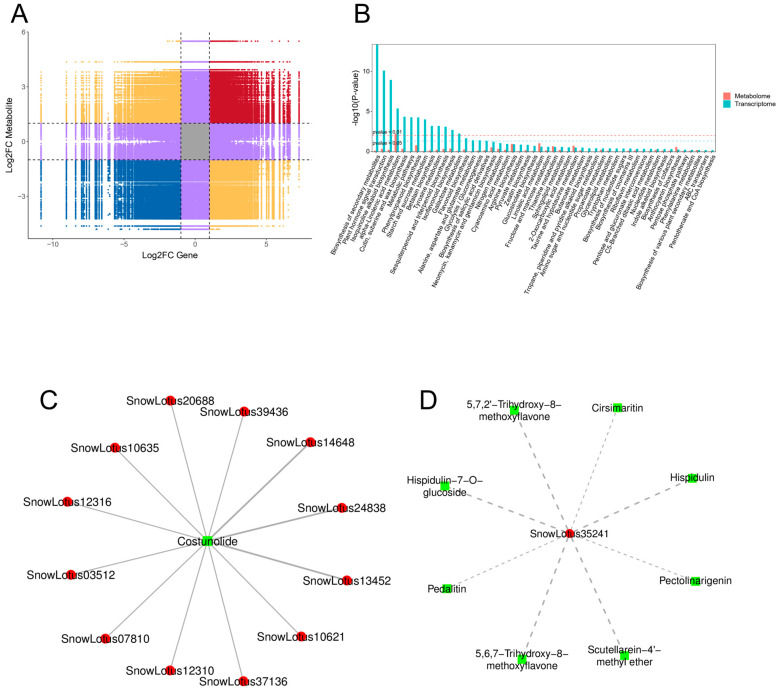
Correlation analysis of DAMs and DEGs. (**A**) Nine-quadrant map of genes and metabolites; (**B**) the co-enrichment of DEGs and DAMs, with blue representing the transcriptome and orange representing the metabolome; (**C**) correlation network diagram for involvement in sesquiterpene and triterpene biosynthesis; (**D**) correlation network diagram for involvement in the biosynthesis of flavones aglycones I, with red denoting genes, green denoting metabolites, solid lines indicating positive regulation, and dotted lines representing negative regulation.

To clarify the association between DEGs and DAMs in *S. involucrata* following various barometric treatments, making up for the problems caused by the limitations of single-omics analysis and the lack of data analysis, we conducted a correlation analysis of the annotation results of DEGs and DAMs within metabolic pathways. This approach enhances the reliability of our findings and provides a clearer understanding of the transcriptional regulatory mechanisms governing these pathways. We screened for correlations that met the criteria of |R| > 0.8 and *p*-value < 0.05. Then, we employed a nine-quadrant diagram to illustrate the differential multiples of genes and metabolites corresponding to these correlation relationships, thereby elucidating the connections between them (Figure 6A). The genes and metabolites in the third and seventh quadrants exhibited consistent differential folds, indicating a stable pattern of expression. This suggests that changes in metabolite expression may be positively regulated by the associated genes. In contrast, the genes and metabolites located in quadrants one and nine displayed opposing differential expression patterns, implying that changes in metabolite expression may be negatively regulated by their corresponding genes.

#### 2.4.2. Key Metabolic Pathways Influenced by Low Pressure

In this study, 123 differential metabolites in the leaves of *S. involucrata* were regulated by 726 differential genes and 74 enriched pathways were identified. The patterns of gene expression for DEGs and DAMs in the metabolic pathways associated with α-linolenic acid, phenylpropanes, and flavonoids showed consistency and significant enrichment. This finding suggests that *S. involucrata* is primarily regulated by these pathways during acclimatization to low-pressure hypoxia. Additionally, we observed a greater number of differential genes enriched in the biosynthesis pathways of isoquinoline alkaloids, sesquiterpenes, and triterpenes. In contrast, a larger number of differential metabolites were enriched in the metabolic pathways of linoleic acid and flavonoid glycoside I (Figure 6B). Notably, in the sesquiterpenoid and triterpenoid biosynthesis (Ko00909), only one metabolite, costunolide, regulated 12 genes, all of which were positively regulated. This suggests that *S. involucrata* is primarily positively influenced by costunolide following low-pressure treatment (Figure 6C, Supplementary Table S4). In contrast, within the biosynthesis of flavone aglycones I (MetMap110) pathway, eight flavonoids, including hispidulin, pedalitin, and 5,6,7-trihydroxy-8-methoxyflavone, negatively regulated the gene SnowLotus35241. This indicates that *S. involucrata* may be negatively regulated by flavonoids after low-pressure treatment (Figure 6D, Supplementary Table S5).

#### 2.4.3. Role of Flavonoids and Phenylpropanoids in Hypoxia Adaptation

**Figure 7:**
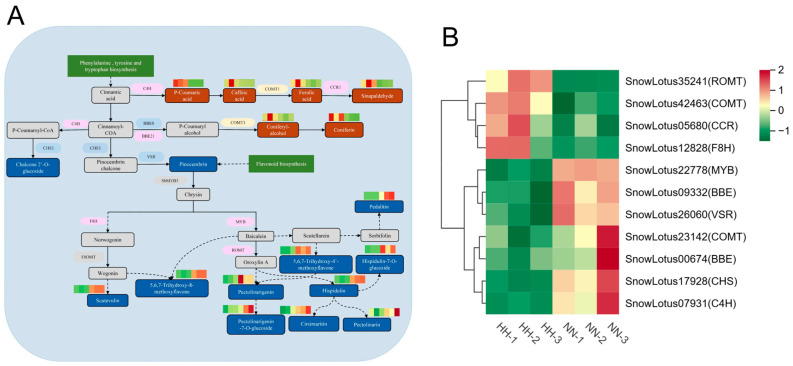
Flavonoid metabolic pathways in response to HH in *S. involucrata*. (**A**) The DEGs and DAMs linked to the flavonoid pathway, where solid lines represent direct regulation and dotted lines indicate indirect regulation. The up-regulated DAMs are highlighted by a red rectangle, whereas down-regulated DAMs are marked with a green rectangle. Up-regulated DEGs are shown in a purple ellipse, down-regulated DEGs are illustrated in a blue ellipse, and bidirectionally regulated DEGs are denoted by a yellow ellipse. (**B**) A clustering heatmap analysis of DEGs related to flavonoid biosynthesis derived from RNA-seq data.

Based on the KEGG enrichment pathway analysis, we identified the DEGs and DAMs involved in the biosynthesis of phenylpropanoids and flavonoids. A flavonoid biosynthesis pathway map was constructed, and DEGs and DAMs were labeled (Figure 7A). Among them, 12 DEGs and 18 DAMs were associated with flavonoid biosynthetic pathways. Flavonoids are a class of plant secondary metabolites derived from phenylpropanoids. Phenylalanine ammonia-lyase (PAL) serves as the initiating enzyme in the phenylpropanoid pathway, while chalcone synthase (CHS) acts as a key enzyme in the metabolic synthesis of flavonoids. A total of five key enzyme DEGs are implicated in phenylpropanoid biosynthesis: one encodes cinnamic acid 4-hydroxylase (C4H), one encodes chalcone synthase 3 (CHS3), two encode caffeic acid 3-o-methyltransferase (COMT), and two encode berberine bridge enzyme (BBE), while one encodes Cinnamoyl-CoA reductase (CCR). PAL and C4H are upstream structural enzymes that play crucial roles in flavonoid biosynthesis. Additionally, four enzymes implicated in flavonoid synthesis were identified: vacuolar-sorting receptor (VSR), flavono-8-hydroxylase (F8H), resveratrol O-methyltransferase (ROMT), and the transcription factor MYB (Figure 7B). There were seven DAMs in the phenylpropanoid biosynthesis pathway. Environments characterized by low pressure and hypoxia can enhance the accumulation of p-coumaric acid, caffeic acid, sinapaldehyde, ferulic acid, coniferyl alcohol, and coniferin. The VSR protein, a membrane protein found in plants, plays a crucial role in the directional transport and degradation of vacuoles within plant cells [29]. The down-regulation of VSR inhibited the levels of pinocembrin, leading to a significant decrease in the concentrations of hispidulin, chalcone-2’-O-glucoside, cirsimaritin, scutevulin, pectolinarin, pectolinarigenin-7-O-glucoside, and 10 other compounds. This effect may be attributed to abnormalities in VSR-mediated vesicle transport that hinder the delivery of these compounds to their intended compartments.

#### 2.4.4. Lipid Metabolism and Stress Response Mechanisms

**Figure 8:**
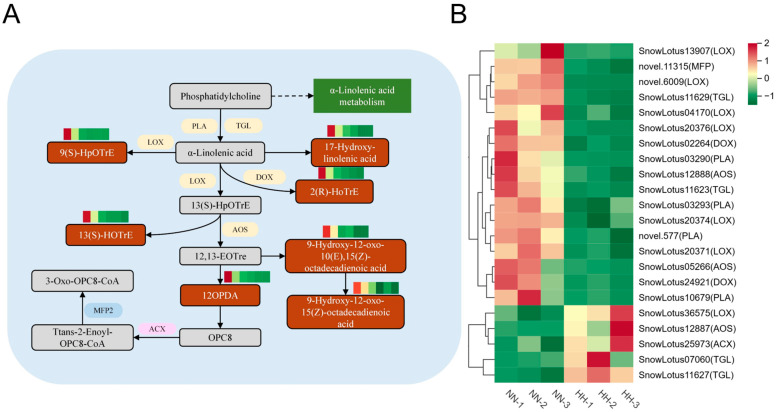
The α-linolenic acid pathway in response to HH in *S. involucrata*. (**A**) DEGs and DAMs associated with the α-linolenic acid pathway. The red rectangle indicates up-regulated DAMs, the purple ellipse denotes up-regulated DEGs, the blue ellipse represents down-regulated DEGs, and the yellow ellipse illustrates bidirectionally regulated DEGs. (**B**) A cluster heatmap analysis of DEGs related to α-linolenic acid metabolism derived from RNA-seq data.

Fatty acids are essential components of cell membranes, and studies have demonstrated that the presence of a kink in the unsaturated fatty acid chain facilitates fluidity. However, when the cell membrane is damaged, this fluidity is impaired, leading to the release of lipids from the membrane due to lipid degradation. α-linolenic acid, a significant unsaturated fatty acid, can be liberated from membrane lipids through the regulation of lipase activity. This acid plays a crucial role in lipid metabolism and is closely associated with plant antioxidants and jasmonic acid (JA) biosynthesis [30]. The KEGG enrichment pathway analysis identified seven DAMs corresponding to seven DEGs within the α-linolenic acid metabolism pathway. Four encode phospholipase A1(PLA), seven encode lipoxygenase (LOX), four encode triacylglycerol lipase (TGL), and two encode dioxygenase (DOX). Three encoded allene oxide synthase (AOS), one encoded acyl-CoA oxidase (ACX), and one encoded multifunctional protein (MFP) (Figure 8B). Among the 22 DEGs, 17 were down-regulated, while 5 were up-regulated. Key genes involved in JA synthesis, such as PLA and AOS, exhibited significant regulation. Additionally, the seven DAMs identified in the alpha-linolenic acid pathway were found to be up-regulated, and LOX was responsible for converting α-linolenic acid into 13(S)-HOTrE, 9(S)-HpOTre, and other unsaturated fatty acids, leading to the production of additional fatty acid hydroperoxides (Figure 8A). OPDA, a key product of JA synthesis, was also found to be significantly up-regulated, and we observed an increase in the content of JA-Ile within the plant hormone transduction pathway.

#### 2.4.5. Identification of Hub Genes and Their Functional Significance

**Figure 9:**
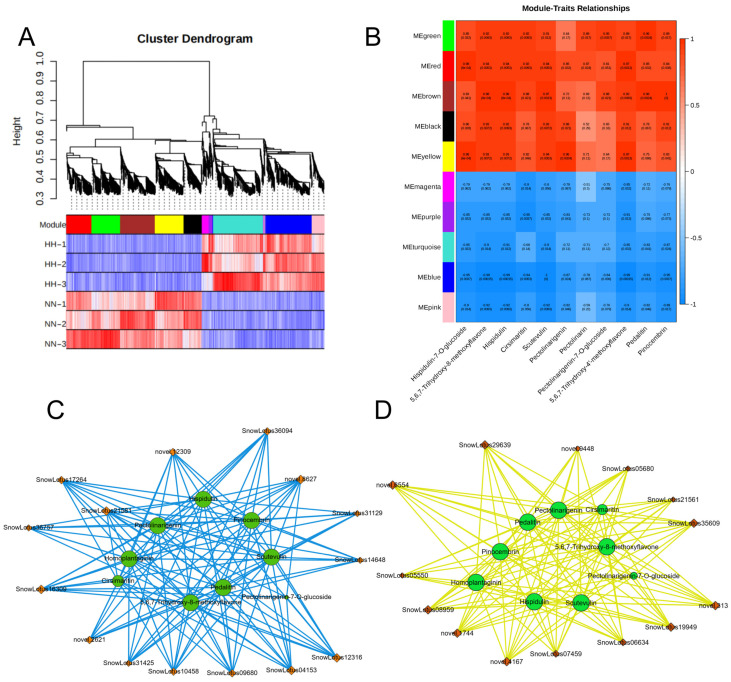
WGCNA analysis of *S. involucrata* genes. (**A**) The results of the cluster tree diagram of the *S. involucrata* DEG expression module, with 10 distinct modules represented in various colors; (**B**) analysis of the relationship between gene modules and 11 flavonoids, with the intensity of the red color indicating a stronger positive correlation, and the intensity of the blue color signifying a stronger negative correlation; (**C**) positive correlation network diagram of hub genes and flavonoids; (**D**) negative correlation network diagram of hub genes and flavonoids, with green circles representing flavonoids, orange diamonds denoting hub genes, blue solid lines indicating promotion of synthesis, and yellow solid lines representing inhibition of accumulation.

To elucidate the gene regulatory network of *S. involucrata* under low-pressure conditions, we conducted a WGCNA utilizing 2383 non-redundant DEGs. This analysis resulted in the identification of 10 distinct modules, each represented by a unique color. Genes within the same module exhibited high correlation coefficients (Figure 9A). Turquoise and blue modules contain 415 and 398 genes, which were positively correlated with HH. Flavonoids were the primary compounds found in *S. involucrata*. Additionally, the phenotypic data of DAMs and DEGs in the flavonoid synthesis pathway were analyzed in relation to module–trait relationships (Figure 9B). The results indicated that flavonoid metabolites exhibited positive correlations with green, red, brown, black, and yellow, while showing negative correlations with magenta, purple, turquoise, blue, and pink. By identifying the three genes with the highest connectivity within each module as hub genes, 15 positive and negative hub genes associated with differential metabolites in the flavonoid pathway were identified (Figure 9C,D). Hispidulin, pectolinarin, and pedalitin were all associated with 15 selected hub genes, whereas cirsimaritin and pectolinarigenin-7-O-glucoside were only related to a subset of these hub genes. The identified hub genes in the KEGG pathway predominantly pertained to plant hormone signal transduction, plant–pathogen interaction, MAPK signaling pathway, and the biosynthesis of sesquiterpenoids and triterpenoids. Consequently, we can infer that these genes may regulate the growth state of *S. involucrata* in low-pressure and hypoxic environments through multiple pathways.

## 3. Discussion

### 3.1. A Multi-Omics Study of Hypobaric Hypoxia Adaptation in S. involucrata

*S. involucrata* is an ancient alpine medicinal plant known for its remarkable pharmacological properties and exceptional stress resistance, yet it is vulnerable to hypobaric hypoxia stress [20]. Research focusing on individual environmental factors has improved our understanding of how low-pressure hypoxic conditions influence the growth and development of *S. involucrata*. However, this approach does not adequately capture the comprehensive effects of various environmental factors present in the plateau environment. This study can only serve as a partial exploration of the adaptation mechanisms of *S. involucrata* in specific low-pressure hypoxic environments. Currently, there is growing evidence that the complex adaptation and evolution of species in hypoxic conditions can be attributed to the synergistic effects of multiple pathways and genes [31]. However, significant gaps remain in the literature regarding the adaptations of high-altitude plants to low-pressure hypoxic environments. In our study, we analyzed the changes in genes and metabolites of *S. involucrata* under normoxic and low-pressure treatments utilizing the transcriptome and the metabolome. This joint analysis provides a unique opportunity to identify candidate genes and metabolites involved in the hypobaric hypoxia pathway of *S. involucrata*. We screened 2383 DEGs and 336 DAMs under low-pressure treatment. The results indicated that the DEGs and DAMs identified at 60 kPa were primarily associated with flavonoids, linolenic acid metabolism, and plant hormones. The regulation of plant responses to hypobaric and hypoxic stress is complex and multifaceted, typically involving multiple response pathways that stabilize the system to adapt to environmental stressors. Our findings indicate that the number of differentially expressed genes and metabolites is down-regulated more frequently than it is up-regulated in HH compared to NN. This suggests that a greater number of genes and metabolites are necessary for the inhibition rather than the activation of *S. involucrata* in low-pressure and low-oxygen environments.

### 3.2. Reprogramming of Flavonoid Metabolism for Hypobaric Hypoxia Acclimation in S. involucrata

The flavonoid synthesis pathway is essential for plant growth and development, encompassing phenylpropanoids, flavonoids, anthocyanins, and isoflavones. This pathway involves structural enzymes, modifying enzymes, transport proteins, and regulatory factors [32]. Flavonoids and phenolic acids are commonly recognized as non-enzymatic antioxidants that help plants manage oxidative stress induced by hypobaric hypoxia environments [33]. Metabolomic analyses revealed that flavonoids such as hispidulin and pectolinarin were significantly down-regulated under low-pressure treatments. In contrast, terpenoids like costunolide and certain phenolic acid metabolites, including 5-O-feruloylquinic acid, exhibited an upward trend in regulation (Figure 5B). The down-regulation of flavonoids, which serve as crucial antioxidants, may indicate a limitation in metabolic resources due to ROS accumulation during the initial phase of hypoxia or a prioritization of essential survival functions by minimizing secondary metabolic consumption. Among flavonoids, anthocyanin-related transcription factors (TFs), particularly MYB, have been extensively studied [34]. In *Ginkgo biloba*, the transcription factor R2R3-MYB is mainly responsible for the negative regulation in the flavonoid biosynthesis pathway [35], and the C2 motif of the MYB, also known as the ethylene response factor (ERF), is recognized as a crucial phytoinhibitory hormone in plants that suppresses flavonoid biosynthesis [36,37]. In our study, we observed that the biosynthesis pathway of flavone aglycones in *S. involucrata* leaves suppressed a decrease in the content of 10 flavonoids, including hispidulin, pectolinarin, and pedalitin, through the significant up-regulation of MYB78. We hypothesize that the C2 motif of MYB is associated with ERF, and that the flavonoid synthesis pathway may regulate MYB under low-pressure hypoxic conditions, thereby indirectly activating the ethylene pathway in response to oxidative stress induced by the low-pressure hypoxic environment.

### 3.3. JA-Mediated α-Linolenic Acid Antioxidant Defense in S. involucrata Under Hypobaric Hypoxia

One of the primary survival strategies employed by plants during hypoxia or hypoxia recovery is the upregulation of antioxidant defense mechanisms, which serve to minimize the occurrence of oxidative stress [38,39]. The signaling pathway involving JA plays a crucial role in regulating the oxidative stress response during reoxygenation, with the JA-mediated reoxygenation response being governed by the transcription factor MYC2 [39]. We integrated transcriptomic and metabolomic analyses to demonstrate that the leaves of *S. involucrata* activate the fatty acid oxidative metabolic pathway of α-linolenic acid metabolism under low-pressure hypoxia treatment. α-Linolenic acid metabolic fluxes may represent the pathway of multiple unsaturated fatty acid biosynthesis [40]. Furthermore, the reaction products of downstream genes such as LOX, AOS, and AOC serve as key enzymes in jasmonate biosynthesis and signaling pathways [41]. In our study, the expression of seven key JA biosynthesis genes, LOX, AOS, ACX, DOX, MFP, PLA, and TGL, was found to be regulated during low-pressure treatment (Figure 8A). This finding suggests that during the low-pressure hypoxia phase, *S. involucrata* may rapidly accumulate JA precursors and activate antioxidant defenses through an increased metabolic flux in α-linolenic acid [39].

### 3.4. Coordinated Regulation of Hypobaric Hypoxia Acclimation in S. involucrata

Heat shock proteins (HSPs) function as molecular chaperones and play critical roles in enhancing plant tolerance to both biotic and abiotic stresses. Research has demonstrated that HSPs in soybean exhibit a significant response to hypoxia stress [42]. In our study, we identified 25 HSPs involved in the protein processing pathway of the endoplasmic reticulum that respond to low-pressure hypoxia treatment (Supplementary Table S6). Furthermore, anaerobiosis has been shown to induce HSPs that overlap with those activated by heat shock and are regulated by heat shock transcription factors, contributing to hypoxia tolerance [43]. Previous studies have demonstrated that Arabidopsis thaliana grown in low-pressure environments of 50 and 25 kPa exhibit a majority of responsive genes localized to metabolic pathways, secondary metabolite biosynthesis, and α-linolenic acid metabolism [44]. These metabolic pathways in *S. involucrata* also proved to be the most representative pathways under hypobaric hypoxia environments. KEGG enrichment analysis indicated that DEGs and DAMs were significantly enriched in the plant hormone signal transduction pathways. These plant hormones encompass a variety of regulatory pathways, including those related to gibberellin, abscisic acid, growth hormone, cytokinin, and ethylene [45]. Ethylene is essential for the hypoxic stress response, and in the absence of oxygen, it enables plants to adapt to their environment primarily by enhancing adventitious root formation, promoting the development of aeration tissues, and increasing petiole and stem height [46]. In this study, we discovered that within the ethylene signaling pathway, EBF1/2 regulates the hypoxia-responsive factors ERF1 and ERF2 by facilitating the catabolism of the EIN3 transcription factor [47]. Growth hormone is also necessary for plant growth and development, and Aux/IAA functions as a transcriptional repressor that interacts with auxin response factors (ARFs) to regulate the expression of early growth hormone-responsive genes [48]. Our study identified 16 DEGs involved in auxin regulation, among which 6 DEGs related to AUX/IAA and Gretchen Hagen (GH3) were found to be up-regulated. IAA initially associates with L-amino acids through the GH3 acylamide synthase family, forming IAA–amino acid conjugates that mediate IAA inactivation. Consequently, the overexpression of the GH3 gene may lead to a reduction in auxin signaling [49]. The signaling pathways associated with various plant hormones do not operate independently in influencing the growth and development of *S. involucrata* in low-pressure environments. Instead, these pathways interact by modulating the expression levels of key genes as a result of integrating different hormonal signals.

## 4. Materials and Methods

### 4.1. Treatment of S. involucrata

In our research, the leaves of *S. involucrata* served as the experimental materials. The seedlings of *S. involucrata* were sliced into small pieces measuring 0.5 cm × 0.5 cm and subsequently placed in the tissue culture induction medium. The resulting tissue culture plantlets were cultured in the medium for over three generations, with each generation lasting 3 weeks for the experiment. Following a one-week acclimatization period in the tissue culture room (temperature: 25 °C; light: 14 h; humidity: 70%), the tissue culture seedlings were transferred to vacuum desiccators maintained at normobaric normoxic (101 kPa) and hypobaric hypoxia (60 kPa) conditions, while other environmental parameters were kept constant. Samples were collected after three weeks. The normobaric normoxic and hypobaric hypoxia treatments were administered three times. The samples were promptly frozen in liquid nitrogen and stored at −80 °C.

### 4.2. RNA Extraction and cDNA Reversal

The *S. involucrata* leaves were subjected to RNA extraction following the protocol of the RNAprep Plant Extraction Reagent (DP441, Tiangen Biotech Co., Ltd., Beijing, China). To assess the integrity of the RNA, it was detected using 1% agarose gel electrophoresis, and total RNA quality was determined by 260/280 UV absorption assay (CYT5M-SN, Agilent Technologies, Santa Clara, CA, USA). One portion of the extracted RNA was designated for real-time fluorescent quantitative PCR, while the other portion was allocated for transcriptome sequencing, which was carried out by Wuhan Biotechnology Company, China. For cDNA synthesis, the aforementioned RNA samples served as templates, and the process adhered to the guidelines of the one-step gDNA removal and cDNA first-strand synthesis kit (AT311, Quanshijin Biotechnology Co., Ltd., Beijing, China). The resulting cDNA was preserved at −20 °C.

### 4.3. Sequence Data Processing and Analysis of DEGs

Total RNA was extracted from the leaves, and 3 biological replicates were conducted for RNA sequencing of both the NN and HH group leaves. A Qubit 4.0 fluorometer, along with an MD enzyme marker, was employed to accurately measure the RNA concentration. Furthermore, a Qsep400 biological analyzer was used to evaluate the RNA integrity value. The poly(A) tail-containing mRNA was enriched with oligo(dT) magnetic beads. Following that, double-stranded cDNA was purified through the use of DNA purification magnetic beads. The purified cDNA then underwent end repair, the incorporation of A tails, and ligation to sequencing junctions. This was succeeded by fragment size selection via DNA purification magnetic beads, and ultimately, PCR enrichment was conducted to generate the final cDNA libraries. These libraries were sequenced using the Illumina platform, resulting in a total of 6 cDNA libraries.

Before conducting data analysis, we utilized fastp [50] to rigorously ensure the quality of the original data. These high-quality data were employed to splice transcripts, thereby enhancing the accuracy of subsequent analyses. When filtering the original data, we removed sequence adapters and eliminated low-quality reads that contained five or more uncertain bases or had over 50% of Qphred scores ≤ 20. Following the assessment of sequencing error rates and GC-content distribution, we obtained clean reads for further analysis and used HISAT2 [51] to align the clean reads with the reference genome, thereby obtaining the positional information of the reads on the reference genome or specific genes. We utilized DESeq2 to conduct differential expression analysis between the sample groups, identifying a set of DEGs in the leaves of *S. involucrata* under various treatment conditions. The DEGs identified were rigorously screened and found to be significantly enriched within KEGG and GO pathways. This determination was based on specific statistical criteria, which included a |log2Fold Change| ≥ 1 and FDR < 0.05.

### 4.4. Metabolite Extraction and DAM Analysis

*S. involucrata* is a high-altitude medicinal plant rich in secondary metabolites. To explore the varied accumulation of metabolic changes triggered by low-pressure hypoxia, 6 samples underwent non-targeted metabolomic analysis. The samples were vortex-mixed for 30 s every 30 min, with this process repeated 6 times. Afterward, the samples were stored at 4 °C overnight prior to centrifugation at 12,000 rpm for 3 min. The supernatant was then carefully aspirated, and the samples were filtered using a microporous membrane (SCAA-104) with a pore size of 0.22 μm, ensuring they were stored in injection vials for UPLC-MS/MS analysis. Utilizing the Metware database, the analysis included the elimination of isotopic signals; repetitive signals associated with K^+^ ions, Na^+^ ions, and NH^4+^ ions; and recurring fragment ion signals linked to other substances of higher molecular weight. Both qualitative and quantitative assessments were performed using the secondary spectral data and the multi-reaction monitoring mode of triple quadrupole mass spectrometry, with the mass spectral information analyzed through the Analyst 1.6.3 software.

The scores for VIP that were generated through the OPLS-DA model utilized the R package. This analytical approach was employed to pinpoint the differential metabolites present in the leaves of *S. involucrata* subjected to a range of treatment conditions. In the analysis involving two groups, differential metabolites were screened based on VIP > 1 and |Log2 FC| ≥ 1. Metabolites were classified as significantly different when they demonstrated a change of more than 2-fold or less than 0.5-fold when comparing the control and experimental groups. Afterward, the metabolites identified were associated with the KEGG Pathway database.

### 4.5. Combined Transcriptome and Metabolome Analysis

To enhance the reliability of our results, we performed a joint analysis of the metabolome and transcriptome. This analysis revealed KEGG pathways annotated by both the transcriptome and the metabolome. KEGG enrichment analyses were conducted using the R language with the ClusterProfiler package (version 4.6.0). We conducted a focused analysis of DEGs and DAMs related to the flavonoid pathway and the metabolism of α-linolenic acid. Furthermore, we executed a heat map clustering analysis of the DEGs involved in this pathway. The clustering heatmaps were generated using the ComplexHeatmap package (version 2.14.0) in R (version 4.2.2). We selected correlation coefficients with absolute values of greater than 0.8 and *p*-values of less than 0.05. Subsequently, we utilized Cytoscape 3.91 [52] software to construct the relevant network, which provided preliminary insights into the regulatory network of *S. involucrata* in response to a hypobaric hypoxia environment (https://cytoscape.org/ accessed on 18 September 2024). DEGs were utilized to construct co-expression network modules using the WGCNA package. The resulting co-expression modules were integrated with characteristic metabolites in the flavonoid pathway. Characteristic gene values for each module were computed and subsequently employed to investigate associations with flavonoid metabolites.

## 5. Conclusions

In our study, the comparison of transcriptome and metabolome analyses under NN vs. HH treatments revealed significant differences in the expression of DEGs associated with hypoxic pathways during the growth and development of *S. involucrata* under low-pressure treatments. The DEGs and DAMs were extensively annotated in relation to secondary compound metabolism, membrane transport, plant hormones, and protein processing. Interestingly, certain genes associated with drought response were involved in low-stress regulation, such as HSPs. Flavonoids predominantly exhibited negative regulatory roles in low-stress environments. We hypothesize that this may be attributed to *S. involucrata*’s unique resource allocation strategy, which involves inhibiting certain flavonoid synthesis to minimize metabolic consumption under hypobaric hypoxia treatment. This strategy is closely linked to the resource constraints present in its high-elevation habitats. Overall, *S. involucrata* appears to respond to hypobaric hypoxia primarily by initiating reprogramming of energy metabolism and activating oxidative stress defense mechanisms, processes that are regulated by numerous genes and metabolites. These results are crucial for elucidating the molecular mechanisms underlying the adaptation of *S. involucrata* to low-pressure hypoxic environments. They will also provide a substantial number of candidate genes for studying the low-altitude introduction and resource conservation of *S. involucrata*. In future work, we will conduct functional verification of the key genes identified in this study to gain a deeper understanding of the regulatory mechanisms by which *S. involucrata* responds to hypobaric hypoxia.

## Data Availability

Data will be made available on request. The raw RNA-seq data (Accession no. PRJNA1218246) were uploaded to NCBI.

## Supplementary Materials

The following supporting information can be downloaded at: https://www.mdpi.com/article/10.3390/genes16030328/s1, Figure S1: RNA gel image of *Saussurea involucrata*; Figure S2: KEGG enrichment column diagram of DEG; Figure S3: GO enrichment column diagram of DEG; Table S1: RNA-seq data analysis of 6 samples; Table S2: A comparison of RNA-seq data from six samples with the genome of *Saussurea involucrate*; Table S3: Significantly up- and down-regulated top five DEG; Table S4: DEGs and DAMs involved in Sesquiterpenoid and triterpenoid biosynthesis; Table S5: DEGs and DAMs involved in Biosynthesis of flavones aglycones I; Table S6: Regulation of HSP in protein processing in endoplasmic reticulum.
