# Supplementary material for: Transcriptome and Metabolome Analysis of Low-Pressure Regulation in Saussurea involucrata Leaves
Source: Genes (Basel). 2025 Mar 11;16(3):328. doi: 10.3390/genes16030328 (PMC11941927; doi:10.3390/genes16030328)

Supplementary data

Table S1: RNA-seq data analysis of 6 samples

Sample	Raw Reads	Clean Reads	Clean Base(G)	Error Rate(%)	Q20(%)	Q30(%)	GC Content(%)
HH-1	58333332	56974938	8.55	0.01	98.48	95.26	44.58
HH-2	47422560	46339022	6.95	0.01	98.43	95.19	44.34
HH-3	42170150	41235696	6.19	0.01	98.57	95.53	44.38
NN-1	50973276	49950866	7.49	0.01	98.36	94.88	44.54
NN-2	49559530	48442650	7.27	0.01	98.45	95.19	44.46
NN-3	49704022	48804414	7.32	0.01	98.51	95.34	44.04

Table S2: A comparison of RNA-seq data from six samples with the genome of *Saussurea involucrata*

Sample	Total Reads	Reads mapped	Unique mapped	Multi mapped
HH-1	56974938	48510303(85.14%)	45796910(80.38%)	2713393(4.76%)
HH-2	46339022	39387002(85.00%)	37402044(80.71%)	2132723(4.27%)
HH-3	41235696	34759864(84.30%)	32794786(79.53%)	2109664(4.35%)
NN-1	49950866	42410454(84.90%)	40277731(80.63%)	2066860(4.23%)
NN-2	48442650	41155761(84.96%)	39046097(80.60%)	1984958(4.28%)
NN-3	48804414	40876541(83.76%)	38809681(79.52%)	1965078(4.77%)

Table S3: Significantly up- and down-regulated top five DEGs

Gene ID	log2FoldChange	pvalue	KEGG	hypothetical protein	regulated
novel.2199	7.295569711	1.83133E-07	K15889	Uncertain protein	up
SnowLotus14972	6.661487088	2.32451E-06	--	Uncertain protein	up
novel.2797	6.648005599	1.27957E-06	--	Uncertain protein	up
novel.432	6.542898918	6.38389E-06	--	Uncertain protein	up
novel.1138	6.4827754	4.55071E-06	K14500	BSK5	up
novel.5883	-10.825982441	5.85744E-19	K03260	POLR2	down
novel.6353	-9.044437871	4.50852E-13	K11593	POLR1	down
novel.2793	-8.394706945	2.39254E-11	K03260	Uncertain protein	down
novel.6352	-7.977974487	6.26921E-10	K03260	Uncertain protein	down
novel.2971	-7.866095286	2.36011E-10	K09571	POLR1	down

Table S4: DEGs and DAMs involved in Sesquiterpenoid and triterpenoid biosynthesis

Gene ID	KEGG_map_gene	Meta ID	Compounds	Class I
SnowLotus10635	ko00909	Hmcp004865	Costunolide*	Terpenoids
SnowLotus37136	ko00909,ko01110	Hmcp004865	Costunolide*	Terpenoids
SnowLotus14648	ko00909,ko01110	Hmcp004865	Costunolide*	Terpenoids
SnowLotus10621	ko00909,ko01110	Hmcp004865	Costunolide*	Terpenoids
SnowLotus20688	ko00909	Hmcp004865	Costunolide*	Terpenoids
SnowLotus13452	ko00909	Hmcp004865	Costunolide*	Terpenoids
SnowLotus24838	ko00909	Hmcp004865	Costunolide*	Terpenoids
SnowLotus07810	ko00909	Hmcp004865	Costunolide*	Terpenoids
SnowLotus12316	ko00909,ko01110	Hmcp004865	Costunolide*	Terpenoids
SnowLotus03512	ko00909	Hmcp004865	Costunolide*	Terpenoids
SnowLotus12310	ko00909,ko01110	Hmcp004865	Costunolide*	Terpenoids
SnowLotus39436	ko00909,ko01110	Hmcp004865	Costunolide*	Terpenoids

Table S5: DEGs and DAMs involved in Biosynthesis of flavones aglycones I

Gene ID	KEGG_map_gene	Meta ID	Compounds	Class I
SnowLotus35241	ko00945	MWSslk237	Hispidulin-7-O-glucoside*	Flavonoids
SnowLotus35241	ko00945	Zmjp004795	5,6,7-Trihydroxy-8-methoxyflavone*	Flavonoids
SnowLotus35241	ko00945	MWSHY0069	5,7,4'-Trihydroxy-6-methoxyflavone*	Flavonoids
SnowLotus35241	ko00945	MWSmce415	4',5-dihydroxy-6,7-dimethoxyflavone)*	Flavonoids
SnowLotus35241	ko00945	MWSHC20114	5,7,2'-Trihydroxy-8-methoxyflavone*	Flavonoids
SnowLotus35241	ko00945	Zmjp006969	Pectolinarigenin*	Flavonoids
SnowLotus35241	ko00945	Lmdp006732	5,6,7-Trihydroxy-4'-methoxyflavone*	Flavonoids
SnowLotus35241	ko00945	Hmcp004865	Pedalitin*	Flavonoids

Table S6: Regulation of HSP in protein processing in endoplasmic reticulum

Gene ID	Swissprot	KEGG_map_gene	log2FoldChange	padj	regulated
SnowLotus00311	HSP	Ko04141	-1.637	5.36589E-26	down
SnowLotus00319	HSP	Ko04141	-1.441	2.2004E-14	down
SnowLotus02032	HSP	Ko04141	-2.098	9.74651E-07	down
SnowLotus06435	HSP	Ko04141	-1.739	1.13408E-24	down
SnowLotus07176	HSP	Ko04141	-1.489	8.27259E-10	down
SnowLotus10392	HSP	Ko04141	-2.862	7.41926E-14	down
SnowLotus12280	HSP	Ko04141	-2.454	1.218E-81	down
SnowLotus17562	HSP	Ko04141	-2.848	0.0098	down
SnowLotus20897	HSP	Ko04141	-1.281	2.25205E-08	down
SnowLotus23230	HSP	Ko04141	-2.720	7.85862E-35	down
SnowLotus23800	HSP	Ko04141	-1.068	2.55651E-12	down
SnowLotus23801	HSP	Ko04141	-2.601	6.76992E-27	down
SnowLotus23910	HSP	Ko04141	-2.091	1.20479E-05	down
SnowLotus32617	HSP	Ko04141	-2.219	1.26892E-48	down
SnowLotus32619	HSP	Ko04141	-2.471	2.95424E-07	down
SnowLotus32624	HSP	Ko04141	-1.372	4.11943E-06	down
SnowLotus32626	HSP	Ko04141	-1.983	2.78238E-05	down
SnowLotus35695	HSP	Ko04141	1.304	0.003307303	up
SnowLotus39111	HSP	Ko04141	-1.280	6.26127E-13	down
SnowLotus39169	HSP	Ko04141	-1.685	3.07109E-21	down
SnowLotus39170	HSP	Ko04141	-2.552	5.58512E-25	down
SnowLotus41075	HSP	Ko04141	-2.273	3.3255E-37	down
SnowLotus41077	HSP	Ko04141	-1.627	1.12219E-22	down
SnowLotus41596	HSP	Ko04141	-2.579	1.12783E-21	down
SnowLotus42113	HSP	Ko04141	-1.393	0.007641333	down

Figure S1: RNA gel image of *Saussurea involucrata*

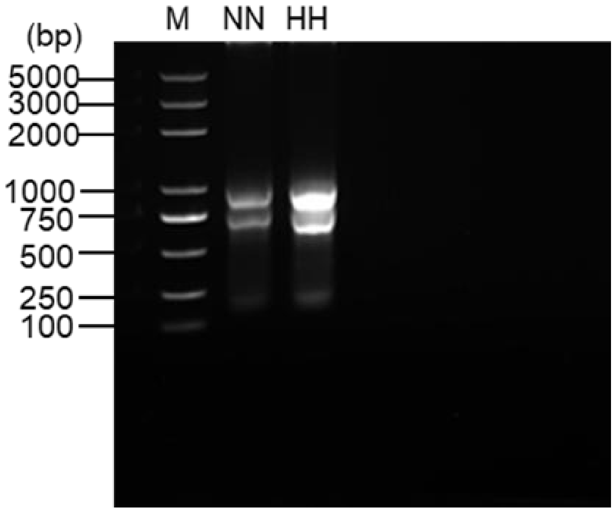

Figure S2：KEGG enrichment column diagram of DEGs

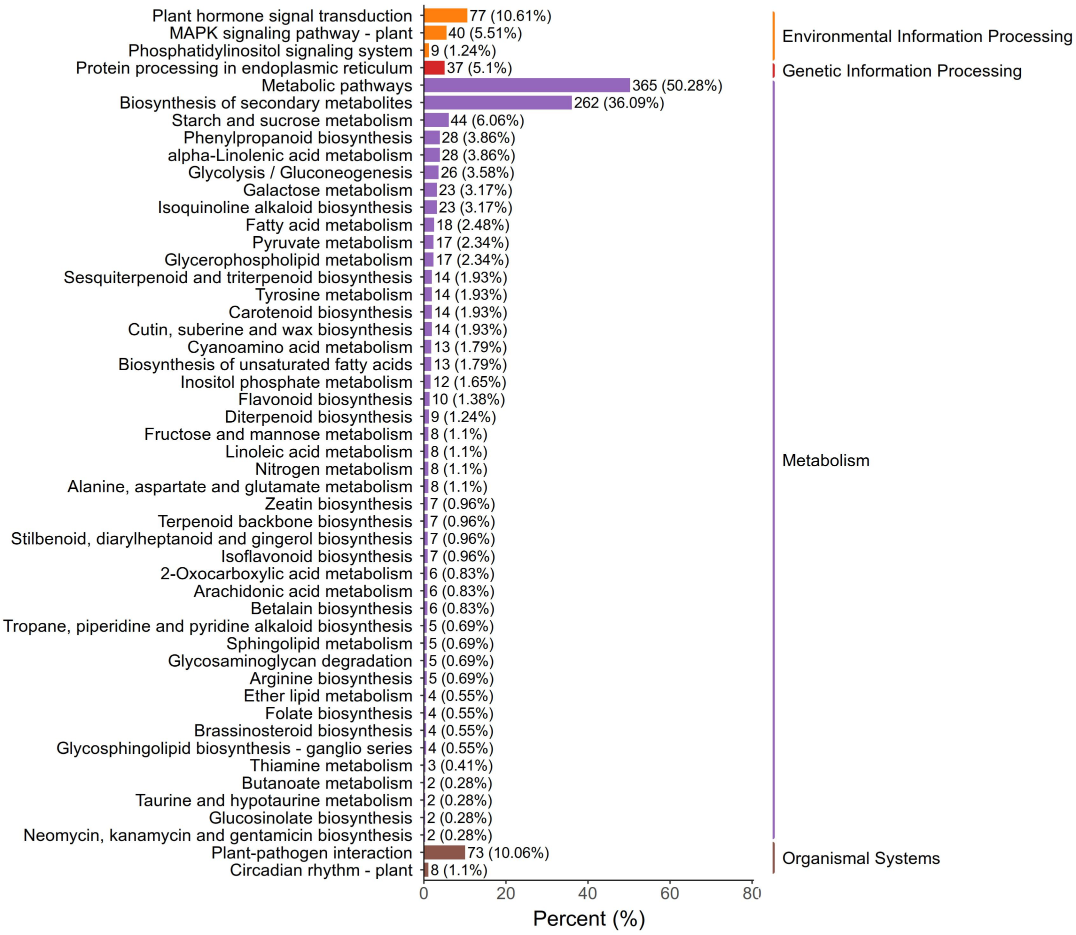

Figure S3：GO enrichment column diagram of DEGs

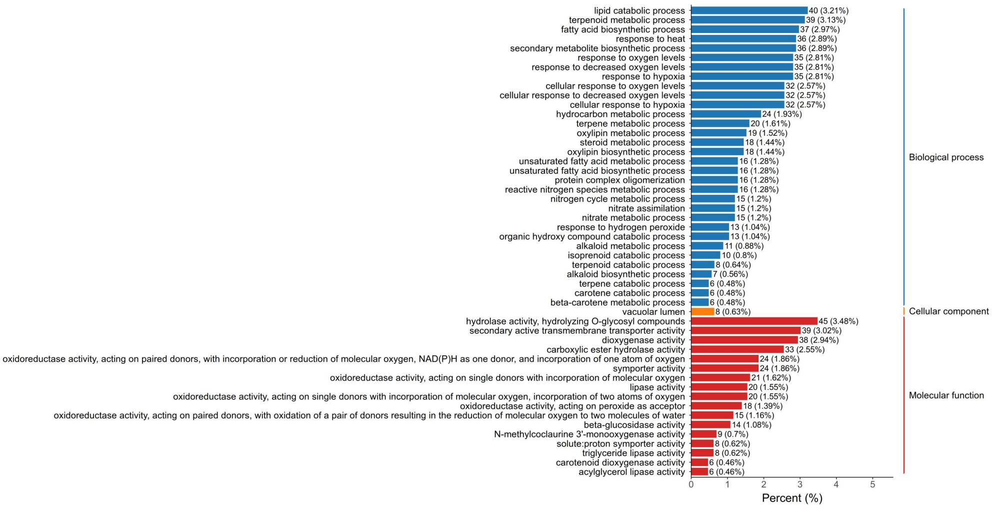

Supplement: Supplementary file 1 [file genes-16-00328-s001.zip › genes-3499611-supplementary.pdf]
